# Prognostic Modeling Integrating *RAS* and *SMAD4* Status in Patients with *BRAF* Wild-Type, Microsatellite-Stable Metastatic Colorectal Cancer

**DOI:** 10.1158/2767-9764.CRC-26-0372

**Published:** 2026-11-10

**Authors:** Vincenzo Nasca, Gabriele Tinè, Henry Walch, Giulia Maddalena, Marco Maria Germani, Filippo Ghelardi, Eleonora Perissinotto, Chiara Boccaccio, Paolo Manca, Matteo Fassan, Matteo Landi, Camilla Damonte, Eleonora Gusmaroli, Alessia Garavaglia, Francesca Bergamo, Chiara Cremolini, Sara Lonardi, Rosalba Miceli, Filippo Pietrantonio, Giovanni Randon

**Affiliations:** 1 Department of Medical Oncology, Fondazione IRCCS Istituto Nazionale dei Tumori, Milan, Italy.; 2 Unit of Biostatistics for Clinical Research, Fondazione IRCCS Istituto Nazionale dei Tumori, Milan, Italy.; 3 Kravis Center for Molecular Oncology, Memorial Sloan Kettering Cancer Center, New York, New York.; 4 Department of Medical Oncology 1, Istituto Oncologico Veneto, Istituto di Ricovero e Cura a Carattere Scientifico, Padua, Italy.; 5 Unit of Medical Oncology 2, Azienda Ospedaliera Universitaria Pisana, Pisa, Italy.; 6 Department of Surgery, Oncology and Gastroenterology, University of Padua, Padua, Italy.; 7 Surgical Pathology and Cytopathology Unit, Department of Medicine-DIMED, University of Padua, Padua, Italy.; 8 Veneto Institute of Oncology, IOV-IRCCS, Padua, Italy.

## Abstract

We aimed to develop clinical–molecular prognostic nomogram for patients with microsatellite-stable (MSS) and *BRAF* wild-type metastatic colorectal cancer (mCRC) using comprehensive genomic profiling and clinical data, taking advantage from a prospectively maintained dataset at three Italian institutions and three randomized trials. The overall cohort was split into development and validation sets. The prognostic model was built on the development set using prespecified landmark intervals. Genomic and clinical predictors were identified within a stability-selection framework based on an L1-penalized Cox model and subsequently incorporated into a stratified Cox model, with metastasectomy modeled as a time-dependent covariate. The overall cohort included 1,071 patients. Stability selection identified *RAS* [stability selection threshold (SST): 98%] and *SMAD4* (SST: 92%) as the only genomic alterations providing additional prognostic information beyond the prespecified clinical model. In the overall population, patients with *RAS*/*SMAD4* double–wild-type tumors had the most favorable overall survival, whereas *RAS* mutations, *SMAD4* alterations, and *RAS*/*SMAD4* co-alterations were associated with inferior outcomes. The final nomogram integrated *RAS*/*SMAD4* with clinical variables and metastasectomy. In the development cohort, time-dependent AUCs were 0.723 and 0.736 at 1 and 4 years, respectively. In the validation cohort, AUCs were 0.657 and 0.711 at the same time points. These findings suggest that prognostically relevant genomic information in MSS, *BRAF* wild-type mCRC may be captured by targeted next-generation sequencing panels including *RAS* and *SMAD4*. By integrating these alterations with clinical variables and time-updated information on metastasectomy, this clinical–molecular nomogram provides individualized dynamic survival estimates.

**Significance:**

*SMAD4* alterations, alongside *RAS* mutations, identify mCRCs with aggressive biology and poor outcomes. By combining readily available clinical and genomic data, the nomogram may support survival estimation and patient counseling. It also provides a reference framework for developing future models as first-line mCRC landscape continues to evolve.

## Introduction

Nomograms are widely used in daily oncology practice as tools for informing patients on prognosis, for guiding treatment-decision algorithms, and for guiding trial design, by integrating multiple clinical–pathologic prognostic factors into statistical models (1). Focusing specifically on patients with metastatic colorectal cancer (mCRC), the recently reported ARCAD nomogram reasonably predicted patients’ prognosis based on patient and tumor characteristics but barely incorporated molecular features (limited to *KRAS* and *BRAF* status only; ref. 2). The widespread adoption of comprehensive genomic profiling (CGP) in clinical practice has generated a large amount of genomic data, which may enhance patient prognostication when combined with clinical variables. In patients with mCRC, several genomic alterations, especially *BRAF*, but also *RAS* and *SMAD4* mutations, *HER2* amplification, *CDKN2A* deletions, and *APC* wild-type status, have been identified as independent negative prognostic factors (3). Therefore, there is a strong rationale for developing innovative molecular nomograms that integrate extended next-generation sequencing (NGS) data with established clinical factors to refine prognostication. Importantly, such tools may be most meaningful when applied to selected subgroups of patients with mCRC, rather than to the overall population. In fact, the molecular heterogeneity of mCRC translates into distinct treatment options and outcomes. In particular, tumors with mismatch repair deficiency and/or high microsatellite instability (MSI-H), as well as those with *BRAF*^V600E^ mutations, should be considered as separate disease subgroups with regard to their intrinsic prognosis and the availability of distinct first-line strategies (4, 5). Based on these premises, we assessed the contribution of CGP to prognostication in a large multicenter prospective cohort of patients with mCRC and developed an integrated clinical–genomic nomogram applicable to patients with proficient mismatch repair (pMMR)/microsatellite-stable (MSS) and *BRAF* wild-type mCRC.

## Materials and Methods

### Patient population

Patients with mCRC undergoing CGP from either the primary tumor or a metastatic site were retrieved from four screening sources: the TRIBE-2 trial (NCT02339116), the PANDA trial (NCT02904031), the ATEZOTRIBE trial (NCT03721653), and the multi-institutional prospective observational study established at three Italian Cancer Centers (Fondazione IRCCS Istituto Nazionale dei Tumori di Milano, Istituto Oncologico Veneto IRCCS, and Azienda Ospedaliero Universitaria Pisana). Key inclusion criteria were pMMR/MSS status, absence of *BRAF* class I (e.g., V600E) or II mutations (6), age ≥18 years, availability of prespecified prognostic baseline clinical features, survival follow-up data, and written informed consent.

Clinical baseline characteristics collected at the time of metastatic disease diagnosis were age, sex, Eastern Cooperative Oncology Group (ECOG) performance status (PS), primary tumor sidedness (right vs. left), primary tumor resection (yes vs. no), time to metastases (synchronous vs. metachronous; synchronous metastatic disease was defined by diagnosis of metastases within 6 months from surgery of the primary tumor or *de novo* diagnosis of metastatic or locally advanced unresectable disease), number of metastatic sites (≥2 vs. 1), and specific sites of metastases. Additionally, information was collected on whether R0/R1 surgical resection of metastases with potentially curative intent had been performed and the date of the procedure.

The study was approved by the Institutional Review Board of the Fondazione IRCCS Istituto Nazionale dei Tumori di Milano (INT 117/15) and was conducted in accordance with the ethical principles for medical research involving human subjects adopted in the Declaration of Helsinki.

### NGS assays

CGP was conducted by FoundationOne CDx assays (F1CDx) in PANDA (7) and ATEZOTRIBE (8) trials and the observational study and by the Caris Molecular Intelligence 592-gene NGS platform in the TRIBE-2 (9) trial, as described previously.

F1CDx was performed by a Clinical Laboratory Improvement Amendments–certified and College of American Pathologists–accredited laboratory (Foundation Medicine, Inc.), which detects gene alterations through at least 324 genes (10). Caris Molecular Intelligence 592-gene NGS was performed at Caris Life Sciences. Overall, the genomic data from both panels were merged, retaining a core set of 324 genes covered by both, whereas excluding the additional genes covered by Caris but not by F1CDx.

Details on the interpretation of gene alterations can be found in Supplementary Methods. Of note, *KRAS* and *NRAS* codon 12, 13, 59, 61, 117, and 146 mutations were grouped together as “typical” *RAS* mutations (in the text, referred hereafter as *RAS* mutations); all other *KRAS* and *NRAS* mutations were classified as “atypical” *RAS* mutations. Variants of uncertain significance as assessed by F1CDx or Caris NGS reports were excluded.

### Statistical analysis

The objective of the study was to develop and validate a prognostic nomogram to predict overall survival (OS) probability in patients with mCRC. OS time was calculated as the interval between the date of diagnosis of metastatic disease to the date of death for any cause, and censoring was applied at the times of last follow-up for patients still alive. Descriptive statistics were used to summarize clinical and biological characteristics. Numerical variables were reported as medians with IQR, whereas categorical variables were described using absolute and relative frequencies. Follow-up time was estimated using the reverse Kaplan–Meier method.

#### Model development

The full cohort was randomly split into development and validation sets in a 3:1 ratio, stratified by calendar period. All steps of the model-building pipeline were conducted in the development cohort. To construct a dynamic prognostic model, landmark intervals were prespecified at baseline (time 0) and every 3 months until 30 months from the diagnosis of metastatic disease. Landmark times were limited to 30 months after metastatic diagnosis to ensure stable estimates and to capture the clinically relevant period in which most metastatic resections and key therapeutic decisions occur. At each landmark, the risk set included only patients alive and uncensored at that time. Postlandmark follow-up used a counting process formulation with left truncation at the landmark and administrative or progressive censoring thereafter.

Model construction proceeded in two stages. First, the NGS panel was reduced by excluding genes with no alterations or with prevalence <1%. Stability selection was subsequently applied within a landmark-stratified Cox model stratified by interval. Clinical variables were specified *a priori* based on established prognostic relevance and included as unpenalized core clinical covariates (age, sex, ECOG PS, primary tumor sidedness, primary tumor resection, time to metastases, number of metastatic sites, and metastasectomy). Candidate genomic predictors (retained gene indicators) and metastatic site variables (lymph node, liver, peritoneal, and lung involvement) were evaluated under penalization and stability selection; metastatic site indicators were penalized to mitigate potential collinearity with the *a priori* number of metastatic site covariate. Genomic information was retained only when consistently selected and when providing incremental prognostic value beyond the *a priori* clinical model. Details on selection procedure are reported in the Supplementary Methods (11). Continuous covariates were modeled with restricted cubic splines with three knots defined in the development cohort to accommodate potential nonlinearity in their relationship with the log-hazard (12). Surgery of metastases was treated as a time-dependent covariate, coded “No” until the date of resection and “Yes” thereafter (right-continuous step function).

Second, the selected predictors were entered into a Cox proportional hazards model stratified by landmark interval, yielding a distinct baseline hazard for each landmark and common regression coefficients across strata. To allow the prognostic effect of surgery of metastases to vary over prediction time, a linear interaction between surgery and landmark time modeled as continuous covariate was specified. Surgery status was assessed at the landmark to ensure a strictly prognostic estimand. Model estimates were reported as hazard ratios (HR) with corresponding 95% confidence intervals (CI).

#### Model validation

Model performance was evaluated both in the development and validation cohort in terms of discriminative ability and calibration for each landmark interval and for prediction horizons of 12, 24, 36, and 48 months after each landmark. Discriminative ability was assessed using the time-dependent AUC, defining cases as patients who died within the time horizon and controls as those alive beyond it. Model estimates were computed using inverse probability of censoring weighting (IPCW) based on the within-stratum Kaplan–Meier estimator of the censoring distribution. Overall prediction error was measured by the dynamic Brier score at the same prediction horizons, also computed with IPCW correction (13). Calibration was assessed using calibration plots at the same horizons and summarized by the dynamic IPCW-corrected Brier score. AUCs and Brier scores were reported at both the stratum-specific and pooled levels (details of pooled metric computation are provided in the Supplementary Methods).

Uncertainty for all performance metrics, stratum-specific and pooled, was quantified using patient-level cluster bootstrap resampling. For stratum-specific metrics, resampling was restricted to patients within the corresponding stratum; for pooled AUC, Brier, and calibration plot, resampling was performed at the patient level over the full cohort, thereby preserving within-patient correlation across landmarks and yielding robust CIs. For each performance metric, 95% CIs were obtained by patient-level bootstrap resampling within each cohort. We also developed a user-facing application to operationalize the landmark Cox framework and facilitate use. All analyses were conducted in R version 4.4.1.

### Validation cohort for prognostic role of candidate genomic predictors

To validate the role of genomic alterations emerging from the stability selection process, we leveraged an external, independent cohort [Memorial Sloan Kettering (MSK)-IMPACT cohort] of samples derived from patients with mCRC profiled with MSK-IMPACT assay (custom NGS panel covering 480 genes; ref. 14). The MSK-IMPACT validation cohort was defined after exclusion of patients for the following reasons: (i) concurrent noncolorectal cancer metastatic malignancies or multiple primary colorectal cancers, (ii) no adequate follow-up, (iii) MSI-H status, (iv) unavailable MSK-IMPACT NGS, and (v) *BRAF* class I/II mutations.

## Results

### Patients and disease characteristics

A total of 1,199 patients with pMMR/MSS mCRC and available CGP receiving first-line treatment with chemotherapy with or without anti-VEGF/EGFR agents were initially screened; after excluding patients carrying *BRAF* class I or II mutations (*n* = 128), 1,071 patients with pMMR/MSS and *BRAF* wild-type mCRC were included in the study. These were subsequently split into development (*n* = 811) and validation (*n* = 260) cohorts (Supplementary Fig. S1). The clinical and biological characteristics of patients stratified by cohort are summarized in Table 1. No major imbalances were observed between the two cohorts. The median follow-up was 64.6 (IQR, 58.5–75.6) and 65.3 (IQR, 58.1–107.5) months for the development and validation cohorts, respectively, whereas the median OS (mOS) was 36.1 (95% CI, 33.6–39.5) and 35.5 (95% CI, 31.3–41) months, respectively (Supplementary Fig. S2).

**Table 1.** Patient and disease characteristics at metastatic disease diagnosis and information on metastasectomy in development (*N* = 811) and validation (*N* = 260) cohorts.

Characteristic	Development *N* = 811 (*n*, %)	Validation *N* = 260 (*n*, %)	*P* value
Age, median (Q1, Q3)	59.9 (51, 67.6)	60.4 (50.9, 68.8)	0.538
Sex	​	​	0.399
Female	347 (42.8)	119 (45.8)	​
Male	464 (57.2)	141 (54.2)	​
ECOG PS	​	​	0.287
0	628 (78.9)	197 (76.7)	​
1	154 (19.3)	51 (19.8)	​
2	11 (1.4)	8 (3.1)	​
3	3 (0.4)	1 (0.4)	​
(Missing)	15	3	​
Primary tumor sidedness	​	​	0.060
Left	562 (69.3)	196 (75.4)	​
Right	249 (30.7)	64 (24.6)	​
Resection of primary tumor	​	​	0.108
Yes	310 (38.2)	85 (32.7)	​
No	501 (61.8)	175 (67.3)	​
Onset of metastases	​	​	0.349
Metachronous	189 (23.3)	68 (26.2)	​
Synchronous	622 (76.7)	192 (73.8)	​
Number of metastatic sites	​	​	0.126
1	449 (55.4)	130 (50)	​
≥2	362 (44.6)	130 (50)	​
Liver metastases	​	​	0.899
Yes	580 (71.5)	187 (71.9)	​
No	231 (28.5)	73 (28.1)	​
Peritoneal metastases	​	​	0.212
Yes	161 (19.9)	61 (23.5)	​
No	650 (80.1)	199 (76.5)	​
Lung metastases	​	​	0.025
Yes	216 (26.6)	88 (33.8)	​
No	595 (73.4)	172 (66.2)	​
Lymph nodal metastases	​	​	0.975
Yes	230 (28.4)	74 (28.5)	​
No	581 (71.6)	186 (71.5)	​
R0/1 metastasectomy	​	​	0.866
Yes	276 (34)	87 (33.5)	​
No	535 (66)	173 (66.5)	​

### Genomic data selection

**Figure 1.**
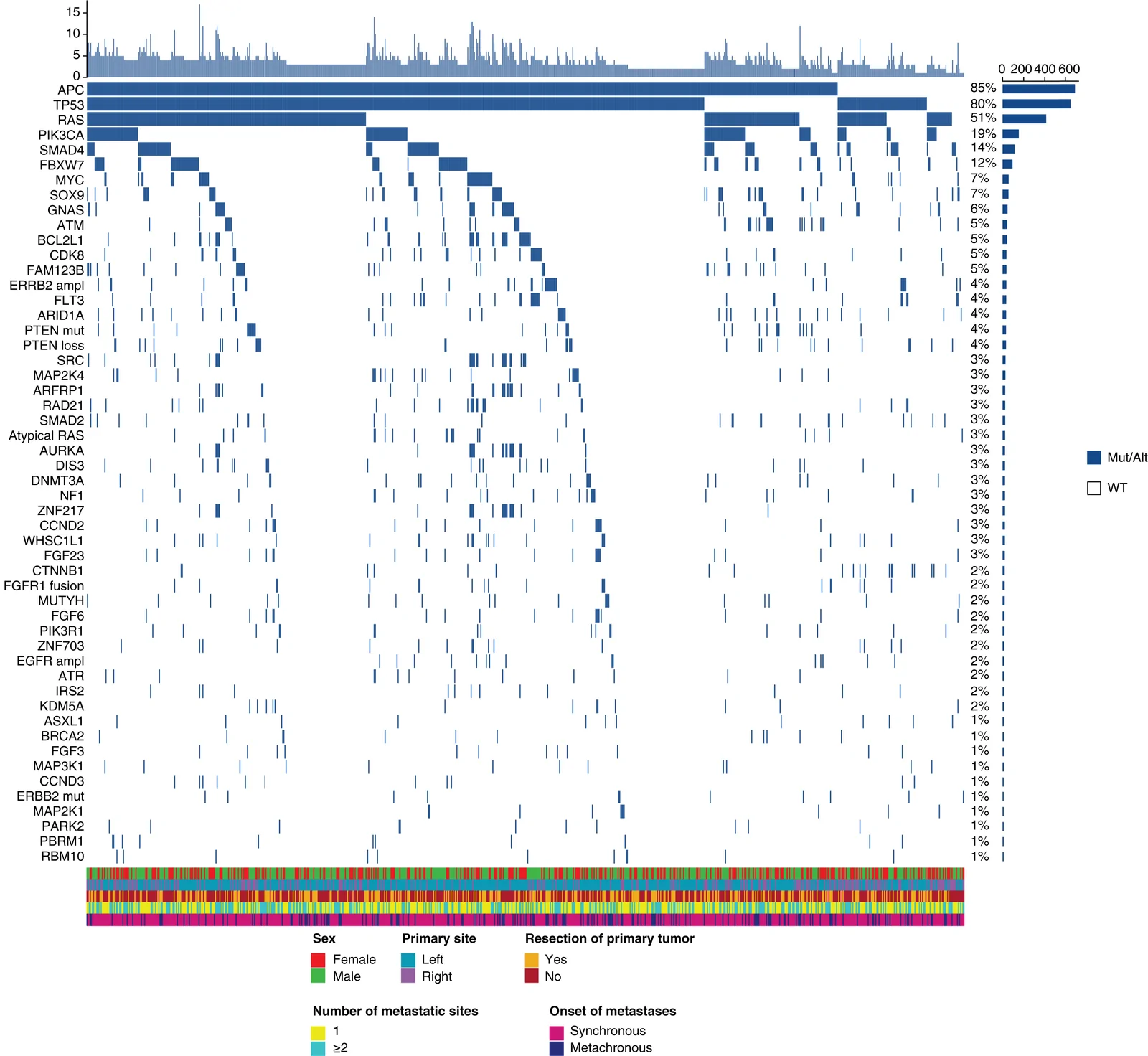
Oncoprint of recurrent genomic alterations from the NGS panel with clinicopathologic annotations. Each column represents one patient and each row a recurrently altered gene; cells are binary-coded as mutated (Mut) vs. wild type (WT). Genes are ordered by decreasing cohort prevalence (percentages shown on right), with *APC* (85%), *TP53* (80%), *RAS* (51%), *PIK3CA* (19%), *SMAD4* (14%), and *FBXW7* (12%) among the most frequent; less common events include *ERBB2* amplification or mutation, *PTEN* mutation or loss, *EGFR* amplification, and *FGFR1* fusion. Clinical annotation tracks indicate sex, primary site (left vs. right), resection of the primary tumor, number of metastatic sites (≥2 vs. 1), and onset of metastases (synchronous vs. metachronous). Columns are arranged to highlight shared and the *x*-axis shows the patient count scale. Alt, altered.

In the development cohort, among the 324 genes assessed by the NGS panels, 91 (28.1%) were never altered and 200 (61.7%) exhibited an alteration prevalence <1% (Supplementary Table S1) and were excluded. The most commonly altered genes were *APC* (85.2%), *TP53* (80.1%), *RAS* (50.9%), *PIK3CA* (19%), *SMAD4* (14.1%), and *FBXW7* (11.6%). Figure 1 displays the mutational pattern across all patients, along with the distribution of the clinical characteristics. At univariable analysis, only four gene alterations were significantly associated with OS: *RAS* (HR, 1.54; 95% CI, 1.32–1.81; *P* < 0.001), *SMAD4* (HR, 1.41; 95% CI, 1.14–1.76; *P* = 0.002), *ATM* (HR, 0.67; 95% CI, 0.46–0.98; *P* = 0.04), and *PIK3R1* (HR, 0.47; 95% CI, 0.24–0.91; *P* = 0.025; Supplementary Fig. S3).

### *RAS*/*SMAD4* status and clinical outcomes

**Figure 2.**
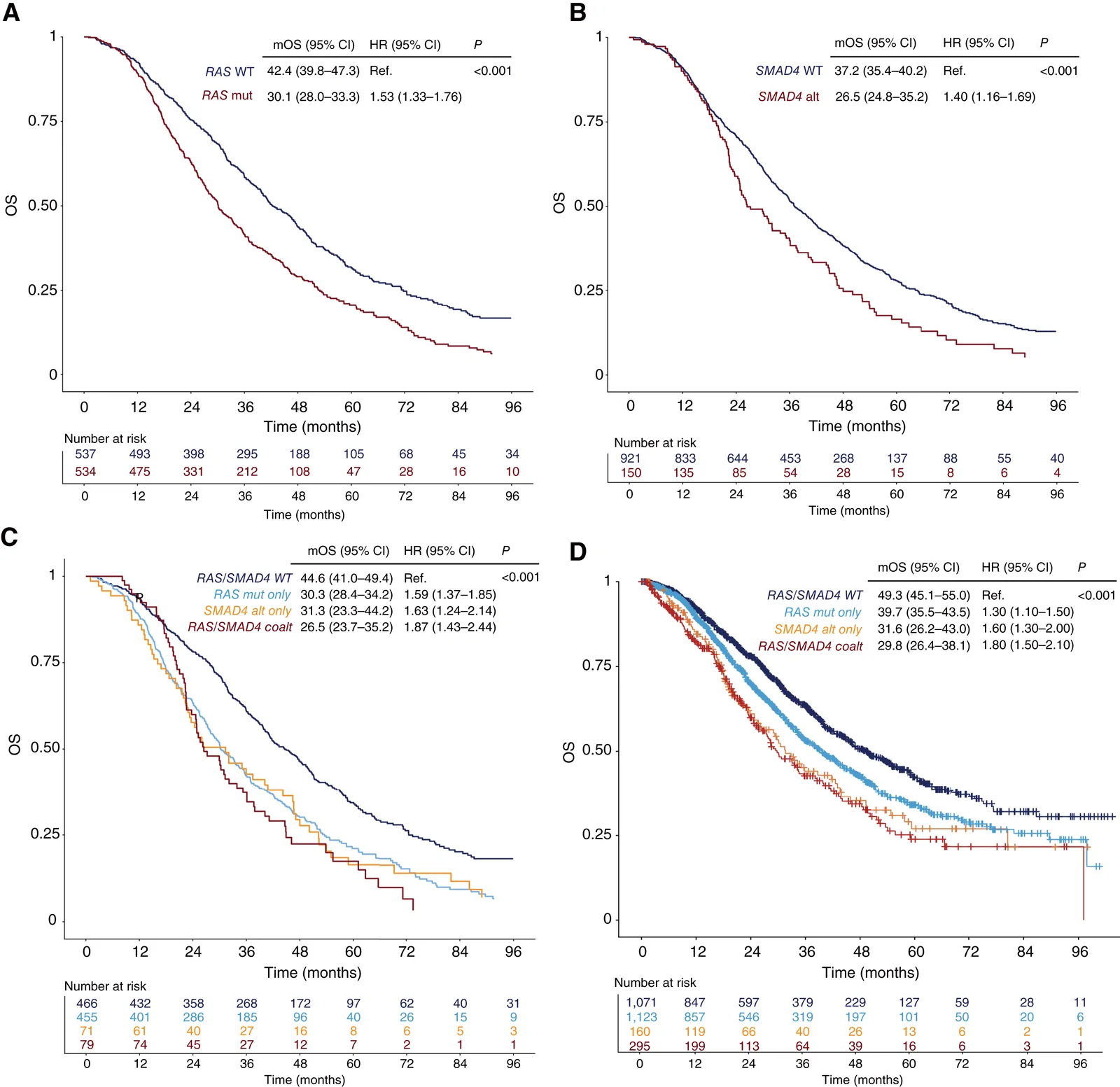
Kaplan–Meier analysis of OS probability according to *RAS* and *SMAD4* status in patients with pMMR/MSS, *BRAF* wild-type mCRC. **A,** Kaplan–Meier OS curves for *RAS* mutational status (wild type vs. mutated) in the overall cohort (*N* = 1,071). **B,** Kaplan–Meier OS curves for *SMAD4* alteration status (wild type vs. altered) in the overall cohort (*N* = 1,071). **C,** Kaplan–Meier OS curves for combined stratification of *RAS* and *SMAD4* status in the overall cohort (*N* = 1,071). **D,** Kaplan–Meier OS curves for combined stratification of *RAS* and *SMAD4* status in the MSK-IMPACT-CRC cohort (*N* = 2,649). Alt, altered; Mut, mutant; Ref., reference; WT, wild type.

In the overall cohort (*N* = 1,071), 466 (43.5%) patients had *RAS*/*SMAD4* wild-type tumors, whereas 455 (42.5%), 71 (6.6%), and 79 (7.4%) had exclusively *RAS* mutations, exclusively *SMAD4* alterations, and concurrent *RAS*/*SMAD4* alterations, respectively. The distribution of clinical factors according to *RAS* and *SMAD4* status is shown in Supplementary Table S2. Briefly, no major imbalances in clinical variable distribution were observed. Kaplan–Meier curves for OS according to *RAS* and *SMAD4* status are shown in Fig. 2A and B. Patients with *RAS*/*SMAD4* double–wild-type tumors had the most favorable survival outcomes (mOS, 44.6 months; 95% CI, 41–49.4). Compared with this reference group, mOS was shorter in patients with *RAS*/*SMAD4* co-altered tumors (mOS, 26.5 months; 95% CI, 23.7–35.2; HR, 1.87; 95% CI, 1.43–2.44), *RAS* wild-type/*SMAD4*-altered tumors (mOS, 31.3 months; 95% CI, 23.3–44.2; HR, 1.63; 95% CI, 1.24–2.14), and *RAS*-mutated/*SMAD4* wild-type tumors (mOS, 30.3 months; 95% CI, 28.4–34.2; HR, 1.59; 95% CI, 1.37–1.85; Fig. 2C). Notably, patients with *RAS/SMAD4* co-altered tumors had a risk of death comparable with that of patients with *SMAD4*-altered tumors (HR, 1.01; 95% CI, 0.59–1.75; *P* = 0.959) but higher than that of patients with *RAS*-mutated tumors (HR, 1.32; 95% CI, 1.03–1.69; *P* = 0.030). To further clarify the negative prognostic value of *SMAD4* alterations, we leveraged an external cohort of 2,649 patients with MSS, *BRAF* wild-type mCRC (MSK-IMPACT-CRC cohort, Supplementary Fig. S4). A total of 1,123 (42.4%), 160 (6%), and 295 (11.1%) patients had *RAS* only, *SMAD4* only, and *RAS*/*SMAD4* co-alterations, respectively. Herein, patients with dual wild-type status achieved an mOS of 49.3 months (95% CI, 45.1–55), whereas those harboring *RAS*, *SMAD4*, or *RAS*/*SMAD4* co-alterations experienced inferior prognosis, with mOS of 39.7 (95% CI, 35.5–43.5), 31.6 (95% CI, 26.2–43), and 29.8 (95% CI, 26.4–38.1) months, respectively (Fig. 2D).

### Nomogram development

Baseline clinical variables and the two genomic alterations emerging from the stability selection process were included in a multivariable landmark Cox model for OS prediction to construct the nomogram. Among all genomic indicators retained after prevalence filtering and jointly evaluated within the penalized, landmark-stratified model, only *RAS* (SST: 98%) and *SMAD4* (SST: 92%) exceeded the prespecified stability-probability threshold of 0.90 and were eventually retained as robust genomic predictors of OS.

The HR estimates (along with 95% CI) of each covariate in the multivariable, landmark-stratified Cox model for OS underlying the nomogram are reported in Table 2. Notably, *RAS* and *SMAD4* alterations were associated with worse OS (HR, 1.24; 95% CI, 1.03–1.50; *P* = 0.022 and HR, 1.41; 95% CI, 1.09–1.81; *P* = 0.009, respectively). Patients who underwent surgical resection of metastases showed a markedly increased OS (HR, 0.36; 95% CI, 0.29–0.44). Additionally, the OS benefit associated with surgical resection of metastases significantly decreased over time (HR of surgery at 6 months, 0.45; 95% CI, 0.37–0.54; HR of surgery at 12 months, 0.56; 95% CI, 0.47–0.67; and HR of surgery at 24 months, 0.81; 95% CI, 0.65–0.97; interaction *P* < 0.001). Other variables associated with significantly worse prognosis included right primary tumor (HR 1.37; 95% CI, 1.11–1.69; *P* = 0.003), primary tumor resection (HR, 1.23; 95% CI, 1.01–1.50; *P* = 0.043), at least two metastatic sites (HR, 1.58; 95% CI, 1.31–1.90; *P* < 0.001), and synchronous metastatic disease (HR, 1.40; 95% CI, 1.12–1.76; *P* = 0.003).

**Table 2.** Multivariable, landmark-stratified Cox model for OS.

Variable	HR (95% CI)	*P* value
Age (years, third vs. first quartile)	​	0.062
67 vs. 50	1.13 (0.99–1.28)	​
Primary tumor sidedness	​	0.003
Right vs. left	1.37 (1.11–1.69)	​
Resection of primary tumor	​	0.043
Yes vs. no	1.23 (1.01–1.50)	​
ECOG PS	​	0.060
≥1 vs. 0	1.25 (0.99–1.57)	​
Number of metastatic sites	​	<0.0001
≥2 vs. 1	1.58 (1.31–1.90)	​
Onset of metastases	​	0.003
Synchronous vs. metachronous	1.40 (1.12–1.76)	​
*RAS* status	​	0.022
Mut vs. WT	1.24 (1.03–1.50)	​
*SMAD4* status	​	0.009
Alt vs. WT	1.41 (1.09–1.81)	​
Surgery of metastases^a^	​	<0.0001
(Yes vs. no) landmark: 0 months	0.36 (0.29–0.44)	​
(Yes vs. no) landmark: 6 months	0.45 (0.37–0.54)	​
(Yes vs. no) landmark: 12 months	0.56 (0.47–0.67)	
(Yes vs. no) landmark: 24 months	0.81 (0.65–0.97)	

HRs with 95% CIs are reported for prespecified clinical and the two selected genomic predictors.

Abbreviations: Alt, altered; Mut, mutated; WT, wild type.

a HRs compare patients who have already undergone metastasectomy and those not yet resected at the specified landmark time. Because the prognostic effect of surgery is modeled through a surgery-by-landmark time interaction term, HRs at any landmark within the 0- to 30-month window can be derived from the fitted model. For parsimony and clinical interpretability, we reported estimates at 0, 6, 12, and 24 months.

**Figure 3.**
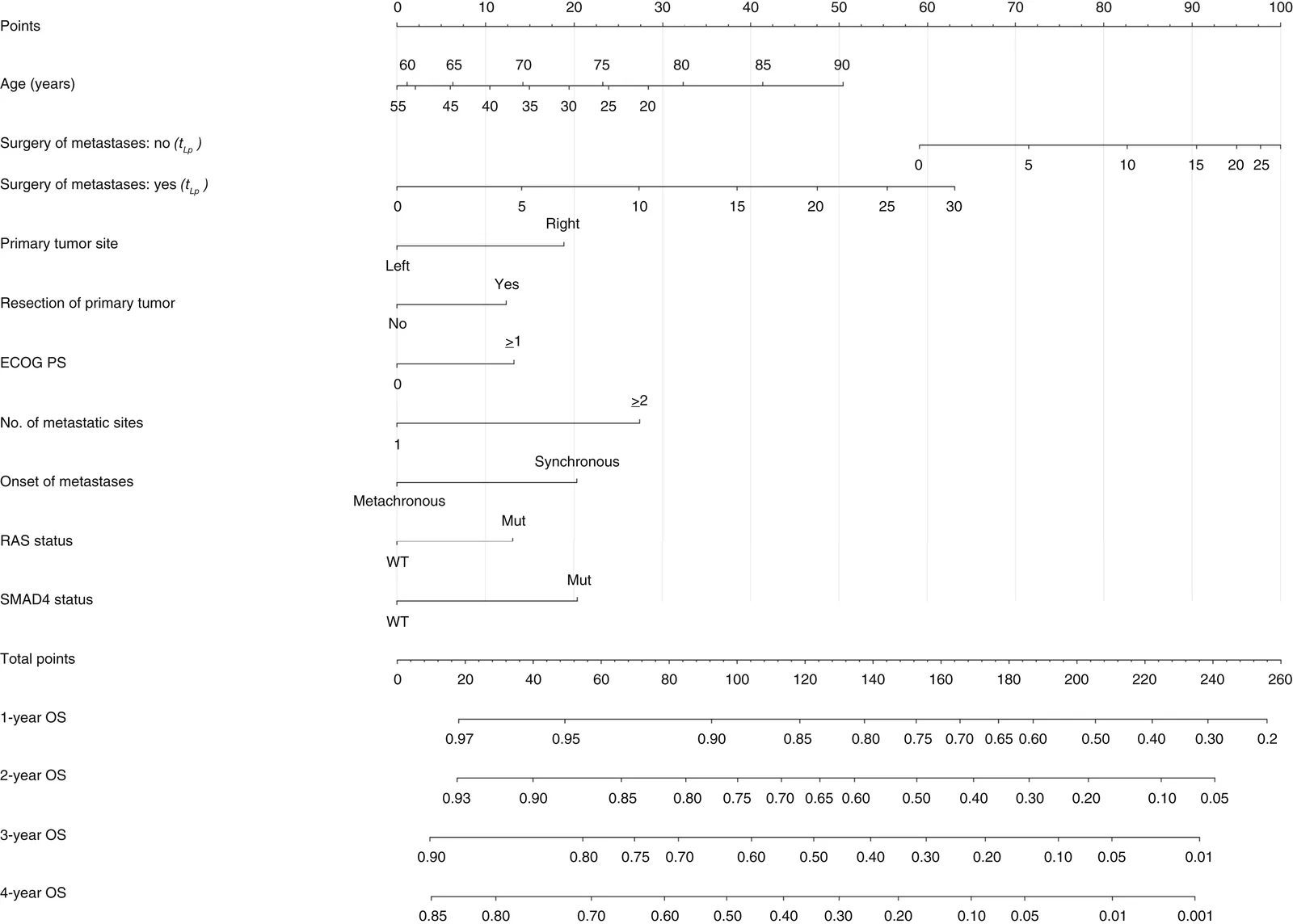
Clinical–molecular nomogram for conditional OS in mCRC. The figure shows the Cox model–derived dynamic nomogram predicting OS probabilities. The nomogram estimates 1-, 2-, 3-, and 4-year OS probability conditional on being alive at a prediction landmark time *t*_*Lp*_ (months since metastatic diagnosis). Landmark times are defined every 3 months from 0 to 30 months. For each *t*_*Lp*_, points are assigned for age, primary tumor site (left vs. right), resection of the primary tumor (yes vs. no), ECOG PS (0 vs. ≥1), number of metastatic sites (1 vs. ≥2), onset of metastases (metachronous vs. synchronous), surgery of metastases (performed vs. not performed), time since surgery (in months; specified only if surgery has been performed), and *RAS* and *SMAD4* status. The total points map to conditional OS probabilities on the lower scales at 1, 2, 3, and 4 years. The effect of surgery of metastases is shown on two lines, “Surgery of metastases: No” and “Surgery of metastases: Yes”. On the “No” line, the horizontal axis represents time since metastatic diagnosis (0–30 months); on the “Yes” line, it represents time since surgery (0–30 months after metastasectomy). *How to use*. First, select a prediction landmark *t*_*Lp*_ (months since metastatic diagnosis). (i) No surgery by the landmark. For a patient who has not undergone metastasectomy by *t*_*Lp*_ (no surgery at all or surgery only after *t*_*Lp*_, i.e., at month *s* > *t*_*Lp*_), use “*Surgery of metastases: No*” line at *t*_*Lp*_, read the points for all covariates, sum them on the *Total Points* axis, and then project vertically to obtain the conditional 1–4-year OS from *t*_*Lp*_. (ii) Surgery before the landmark. For a patient who underwent metastasectomy at month *s* ≤ *t*_*Lp*_, the predictions depend on the time since surgery, i.e., Δ = *t*_*Lp*_ − s. Use “*Surgery of metastases: Yes*” line at Δ, read the points for all covariates, sum them to *Total Points* axis, and project to the survival scales to obtain the conditional 1- to 4-year OS from *t*_*Lp*_. This construction provides dynamically updated, landmark-conditional predictions that reflect both whether surgery has occurred and the time elapsed since surgery.

The nomogram derived from the landmark model is represented in Fig. 3. It includes baseline clinical variables and an additional interaction term between the time-dependent variable corresponding to the surgery of metastases, as well as the mutational status of *RAS* and *SMAD4*, the landmark time as continuous variable, and the 11 strata for each landmark interval. At a clinician-selected landmark (0–30 months since metastatic diagnosis), the nomogram estimates the OS probability at 12, 24, 36, and 48 months in two different scenarios: (i) patients who did not receive resection of metastases at that time point, using baseline covariates and (ii) patients after metastasectomy, explicitly incorporating the timing of surgery relative to the landmark. All predictions are derived from the landmark forward and are conditional on surviving up to that time.

### Model calibration and validation

The pooled performance of the prognostic model across landmarks is summarized in Supplementary Table S4. In the development cohort, time-dependent AUCs indicated good and stable discrimination at each horizon (0.723; 95% CI, 0.70–0.75 at 1 year and 0.736; 95% CI, 0.70–0.77 at 4 years). Corresponding IPCW Brier scores supported good calibration and low prediction error (0.156; 95% CI, 0.15–0.17 and 0.188; 95% CI, 0.17–0.21, respectively). In the validation cohort, pooled AUCs were 0.657 (95% CI, 0.60–0.71) at 1 year and 0.711 (95% CI, 0.63–0.77) at 4 years, showing moderate discrimination at 1 year that improved to good by 4 years. The corresponding IPCW Brier scores were 0.169 (95% CI, 0.15–0.19) at 1 year and 0.192 (95% CI, 0.16–0.23) at 4 years, supporting low prediction error. Additionally, calibration curves for pooled predictions at each time horizon (Supplementary Fig. S5) showed good agreement between predicted and observed survival probabilities. Landmark-specific estimates were consistent with pooled results. In the development cohort, AUCs ranged from 0.671 to 0.751 (1 year), 0.700 to 0.748 (2 years), 0.706 to 0.739 (3 years), and 0.721 to 0.750 (4 years; Supplementary Fig. S6A–S6D). The IPCW Brier scores ranged from 0.080 to 0.197, 0.192 to 0.225, 0.201 to 0.226, and 0.173 to 0.215, at 1, 2, 3, and 4 years, respectively (Supplementary Fig. S7A–S7D). In the validation cohort, AUCs ranged from 0.590 to 0.698, 0.618 to 0.699, 0.602 to 0.736, and 0.628 to 0.793 across the same horizons (Supplementary Fig. S6E–S6H), with Brier scores 0.087 to 0.210, 0.208 to 0.241, 0.210 to 0.252, and 0.152 to 0.231 (Supplementary Fig. S7E–S7H). Variability across landmarks was low, without systematic decline at later landmark times. Supplementary Table S3 reports a direct comparison between the clinical-only and final clinical–molecular models. Addition of *RAS* and *SMAD4* was consistently associated with modest improvements in discrimination and prediction error across all prediction horizons in both cohorts. Across the 1- to 4-year prediction horizons, the absolute increase in time-dependent AUC ranged from 0.010 to 0.021, whereas the absolute reduction in Brier score ranged from 0.004 to 0.007. Nested model comparison also supported their incremental contribution (likelihood ratio test, *P* < 0.001; ΔAIC = −335.6).

### Model application

The nomogram provides time-updated, conditional OS estimates at prespecified horizons. To illustrate the use of the dynamic nomogram application, we present three scenarios for a patient with identical baseline covariates. First, at diagnosis of metastatic disease (month 0), the application returns conditional survival from that starting point (Supplementary Fig. S8). Second, if the same patient undergoes metastasectomy at month 3, the prediction origin is reset to that time of surgery, yielding postsurgical estimates that reflect the change in surgical status (Supplementary Fig. S9). Third, selecting a later postsurgery landmark at 6 months after metastatic diagnosis updates the estimates again by conditioning on having remained alive and under follow-up to that time (Supplementary Fig. S10). These examples are intended to facilitate interpretation of the model; individual estimates can be obtained by assigning the corresponding covariate values and landmark information using the nomogram presented in Fig. 3. These examples show how predictions evolve with clinical events and elapsed time under the landmarking framework and how the application supports clear, bedside “what-if” exploration without reestimating the model.

## Discussion

In this study, *RAS* and *SMAD4* were the only genomic alterations consistently selected as providing prognostic information beyond the prespecified clinical model, and patients harboring either alteration had worse prognosis than those with *RAS*/*SMAD4* double–wild-type tumors. In fact, *RAS* and *SMAD4* alterations are well-known adverse prognostic variables in metastatic disease, with solid biological plausibility (15, 16).

The limited contribution of remaining genomic alterations may have several explanations. First, among the recurrently mutated genes, only a minority exerts a well-defined prognostic role. *APC* alterations have been previously associated with improved survival, especially when involving the N-terminal side compared with truncating alterations involving the C-terminal (17). In our study, *APC* prognostic value was not retained in the final model. This may be partly related to their strong association with *BRAF* wild-type status. Also, *TP53* mutations were not significantly associated with survival, in line with the existing literature data, which show controversial results on their prognostic value (18). Second, only a small subset of genes was altered in a considerable proportion of patients. Therefore, it cannot be excluded that individual genes, such as *CCND3*, might have contributed to refining prognostic stratification had they been altered in a larger proportion of patients. Third, grouping mutations involved in the same pathway may provide more robust prognostic data. Specifically, alterations affecting the DNA damage response, such as those in *ATM* or *BRCA*, or broader mutational patterns, may have greater prognostic relevance than individual genomic alterations (19). However, grouping genes into genomic signatures is often arbitrary, as perturbations of complex pathways are not necessarily driven by single-gene mutations.

Importantly, the addition of *RAS* and *SMAD4* significantly improved model fit (likelihood ratio *P* < 0.001), whereas direct comparison between the clinical-only and clinic–molecular models showed quantitatively modest gains in discrimination and prediction error. Therefore, the nomogram provides a parsimonious way of refining individual prognostication beyond clinical variables, whereas indicating that most prognostic information is captured by clinical and disease-related factors. In fact, recent nomograms in patients with mCRC highlighted that a substantial proportion of prognostic information is contributed by patient- and disease-related factors, including sex, ECOG PS, low body mass index (BMI), or albumin levels (2), with similar data reported for other cancer types, including lung and breast cancers (20, 21). In the ARCAD nomogram, poorer clinical conditions, young or old age, and higher tumor burden were associated with worse OS in a cohort of more than 22,000 patients with mCRC enrolled in first-line clinical trials, in line with our findings (2). Additionally, surgical resection of metastases, particularly in the liver and lungs, represents the single most decisive factor for long-term survival and potential cure in selected patients with mCRC. Given its strong association with survival, incorporating metastasectomy as a time-dependent variable may have limited an additional discrimination from genomic predictors.

This study included resection of metastases as time-dependent variable and may estimate postsurgical survival estimates. However, this model should not be used as a decision-making tool to determine surgical eligibility or to exclude patients from surgery based on predicted outcomes. In fact, surgical resection of metastases is determined by a complex interplay between the initial tumor burden, number of organs involved, and response to systemic treatment, especially for patients with potentially resectable disease or for those candidate to secondary resection of metastases after systemic treatment (22). Indeed, because of the observational and nonrandomized design of the study, as well as the inclusion of patients with a heterogeneous presentation of oligometastatic disease, detailed data about upfront resectability were not available. Consequently, the resulting nomogram should be interpreted with caution, as the underlying dataset includes a mixed population of patients, those who were upfront resectable, those who became resectable after chemotherapy, and those who remained unresectable. With regard to this issue, several studies have explored the risk of disease progression after liver metastasectomy in high-risk subgroups to identify patients less likely to benefit from surgery, such as those with initially unresectable liver metastases (23), those with *BRAF*^V600E^-mutated tumors (24), or those experiencing progression in the interval between chemotherapy and surgery (25). Although these selected case series demonstrated a significant risk of early recurrence or progression, they did not rule out a survival benefit at the individual patient level. Therefore, surgical decision-making should always occur within a multidisciplinary board, taking into account both patient- and tumor-related characteristics as well as dynamic variables such as treatment response. Such complexity and heterogeneity cannot be fully captured by a single prognostic nomogram.

Then, our data suggest that genomic information relevant to prognostication may be captured by small NGS panels, including *RAS* and *SMAD4*. Beyond prognostication, the role of CGP remains crucial for the identification of rare genomic drivers of resistance to targeted, therapeutic targets. Indeed, its utility in refining patient selection for selected targeted therapies (e.g., anti-EGFR, anti-HER2, and anti-BRAF) has been largely investigated. Moreover, CGP may help to identify rarer but potentially targetable alterations (e.g., *POLE*, *ALK*, and *ROS1*), for which specific treatment could be accessible through standard practice or dedicated clinical trials (26). Besides therapeutic decisions, CGP may also provide useful information on hereditary risk and advice on genetic counseling. Additionally, it must be considered that extensive NGS can accomplish these multiple aims with a single panel test, which may be more cost-effective and less tissue wasting than running multiple individual companion diagnostics.

We acknowledge that our work has several limitations. First, its retrospective, multicenter design may introduce selection bias. Second, data on CGP come from two different platforms (Foundation Medicine and Caris), but, given the limited set of genes presenting genetic abnormalities and the relatively low allele frequency of the alterations, it is unlikely that Caris-only NGS would further the prognostic model. Furthermore, other molecular factors, at chromosomal (e.g., copy-number changes) or at transcriptional level, may sharpen prognostic ability of our nomogram, but these data were not available. Third, several prognostic variables (e.g., BMI or laboratory measures of inflammatory state such as albumin or neutrophil-to-lymphocyte ratio) were not consistently available across the contributing datasets and therefore could not be incorporated, which may have limited the model’s discriminative performance and also impaired direct comparability with more data-intensive clinical nomograms (e.g., the ARCAD model). Fourth, we did not collect data on treatment lines and regimens, as it was beyond our scope on prognosis prediction, but therapeutic heterogeneity may influence realized outcomes. Future studies should therefore evaluate treatment-specific models, particularly as molecularly selected first-line strategies become more widely used.

The strengths of our model include its large cohort with patients from both the clinical practice and different phase II/III trials, with splitting of cohort into a training and validation one, ensuring high data quality, fidelity, and reproducibility. Second, we leveraged an external independent cohort of 2,649 patients, which strengthened our findings on the prognostic role of *SMAD4* status, independently of *RAS*. The ongoing clinical development of TGFβ pathway inhibitors provides an additional rationale for investigating *SMAD4* within baseline genomic profiling, beyond its established adverse prognostic significance. In particular, given the central role of SMAD4 in the TGFβ signaling pathway, further research is needed to clarify whether *SMAD4* alterations may serve as predictive biomarkers for TGFβ-targeted strategies, the clinical development of which has been so far limited by modest efficacy and toxicity, although novel agents such as TGFβR2/PD-1 bispecific antibodies may become promising alternatives (27, 28). Then, by including data also on metastases resection and genomic features, assessed through validated NGS techniques, it overcomes limitations of earlier models, making it a contemporary tool for refining the individual prognostication of patients with MSS, *BRAF* wild-type mCRC. Lastly, the model showed moderate but consistent discrimination across landmarks and prediction horizons, with good calibration. This level of performance is broadly consistent with prior prognostic tools in mCRC, supporting clinical utility despite the intrinsic heterogeneity of the disease.

In summary, we developed an easy-to-use, well-calibrated, dynamic nomogram that combines parsimonious genomic data, namely *RAS* and *SMAD4*, with readily available clinical variables to deliver time-conditional survival estimates in pMMR/MSS, *BRAF* wild-type mCRC. Our clinical–molecular nomogram may represent a reference framework for the development of future prognostic models as the landscape of first-line treatment of mCRC evolves. Larger and more contemporary series will likely be needed to derive molecularly stratified, treatment-specific nomograms in populations with existing tailored first-line options (e.g., *BRAF*^V600E^, *HER2* amplification, and *KRAS*^G12C^).

## Supplementary Material

Supplementary Methods Supplementary Methods

Supplementary Figure S1 Supplementary Figure S1. Study flow diagram.

Supplementary Figure S2 Supplementary Figure S2. Overall survival Kaplan-Meier curves, stratified for development and validation cohorts. P-value is from a two-sided log-rank test.

Supplementary Figure S3 Supplementary Figure S3. Univariable Cox model association between overall survival and individual gene alterations from Comprehensive Genomic Profiling after filtering.

Supplementary Figure S4 Supplementary Figure S4. Diagram detailing patient inclusion for BRAF wild-type MSK-IMPACT-CRC cohort (N=2649).

Supplementary Figure S5 Supplementary Figure S5. Calibration plots of pooled overall survival (OS) predictions at 1, 2, 3, and 4 years, obtained by weighted pooling of landmark-specific predictions in the development (A-D) and validation (E-H) cohorts.

Supplementary Figure S6 Supplementary Figure S6. Time-dependent discriminative ability measure (Area Under the Curve [AUC]) at 1-, 2-, 3-, and 4-year horizons across 11 prespecified landmark times (0-30 months, 3-month steps) in the development cohort (panels A-D) and the validation cohort (panels E-H). Each point represents the landmark-specific AUC with the corresponding 95% CI.

Supplementary Figure S7 Supplementary Figure S7. Dynamic prediction error (Brier score) at 1-, 2-, 3-, and 4-year horizons across 11 prespecified landmark times (0–30 months, 3-month steps) in the development cohort (panels A–D) and the validation cohort (panels E–H).

Supplementary Figure S8 Supplementary Figure S8. Dynamic clinic-molecular nomogram to predict overall survival probability for patients at the time of metastatic disease diagnosis (i.e., landmark time origin: month 0).

Supplementary Figure S9 Supplementary Figure S9. Dynamic nomogram to predict overall survival probability for patients undergoing metastasectomy at 3 months from metastatic disease diagnosis (i.e., landmark time origin: month 3).

Supplementary Figure S10 Supplementary Figure S10

Supplementary Table S1 Supplementary Table S1. Frequency of genomic alterations detected by the Comprehensive Genomic Profiling (CGP).

Supplementary Table S2 Supplementary Table S2. Patients and disease’s characteristics according to RAS and SMAD4 status in the overall cohort (N=1071).

Supplementary Table S3 Supplementary Table S3. Pooled performance comparison between the clinical-only landmark Cox model and the final clinic-molecular landmark Cox model including RAS and SMAD4.

## Data Availability

The data generated during and/or analyzed during this study, including genomic data, can be requested; scientific agreements would be required, and the corresponding author can provide the raw data upon reasonable request.
