# Supplementary material for: Prognostic Modeling Integrating RAS and SMAD4 Status in Patients with BRAF Wild-Type, Microsatellite-Stable Metastatic Colorectal Cancer
Source: Cancer Res Commun. 2026 Nov 10;6(11):2644–54. doi: 10.1158/2767-9764.CRC-26-0372 (PMC13750249; doi:10.1158/2767-9764.CRC-26-0372)
Supplement: Supplementary file 2 [file crc-26-0372_supplementary_figure_s1_suppsf1.docx]

**Supplementary Figure S1.** Study flow diagram.

List of Abbreviations. NGS: next-generation sequencing. pMMR: mismatch-repair proficient. MSS: microsatellite stable. CGP: comprehensive genomic profiling. mCRC: metastatic colorectal cancer.
