# Supplementary material for: Prognostic Modeling Integrating RAS and SMAD4 Status in Patients with BRAF Wild-Type, Microsatellite-Stable Metastatic Colorectal Cancer
Source: Cancer Res Commun. 2026 Nov 10;6(11):2644–54. doi: 10.1158/2767-9764.CRC-26-0372 (PMC13750249; doi:10.1158/2767-9764.CRC-26-0372)
Supplement: Supplementary file 3 [file crc-26-0372_supplementary_figure_s2_suppsf2.docx]

**Supplementary Figure S2.** Overall survival Kaplan-Meier curves, stratified for development and validation cohorts. P-value is from a two-sided log-rank test.
