# Supplementary material for: Prognostic Modeling Integrating RAS and SMAD4 Status in Patients with BRAF Wild-Type, Microsatellite-Stable Metastatic Colorectal Cancer
Source: Cancer Res Commun. 2026 Nov 10;6(11):2644–54. doi: 10.1158/2767-9764.CRC-26-0372 (PMC13750249; doi:10.1158/2767-9764.CRC-26-0372)
Supplement: Supplementary file 4 [file crc-26-0372_supplementary_figure_s3_suppsf3.docx]

**Supplementary Figure S3.** Univariable Cox model association between overall survival and individual gene alterations from Comprehensive Genomic Profiling after filtering. Alteration types are indicated in the gene labels (e.g., mut, ampl, loss, fusion). The list of genes is ordered by decreasing Cox model-derived C-index (left panel) and for each gene we show the hazard ratio (HR) comparing altered versus wild-type status and the corresponding two-sided Wald p value (right panel). C-index values of ~0.50 indicate no discrimination; HR>1 (<1) denotes higher (lower) risk of death for altered versus wild-type status.

List of Abbreviations. Mut: mutation. Ampl: amplification.
