# Supplementary material for: Prognostic Modeling Integrating RAS and SMAD4 Status in Patients with BRAF Wild-Type, Microsatellite-Stable Metastatic Colorectal Cancer
Source: Cancer Res Commun. 2026 Nov 10;6(11):2644–54. doi: 10.1158/2767-9764.CRC-26-0372 (PMC13750249; doi:10.1158/2767-9764.CRC-26-0372)
Supplement: Supplementary file 5 [file crc-26-0372_supplementary_figure_s4_suppsf4.docx]

**Supplementary Figure S4.** Diagram detailing patient inclusion for *BRAF* wild-type MSK-IMPACT-CRC cohort (N=2649).
