# Supplementary material for: Prognostic Modeling Integrating RAS and SMAD4 Status in Patients with BRAF Wild-Type, Microsatellite-Stable Metastatic Colorectal Cancer
Source: Cancer Res Commun. 2026 Nov 10;6(11):2644–54. doi: 10.1158/2767-9764.CRC-26-0372 (PMC13750249; doi:10.1158/2767-9764.CRC-26-0372)
Supplement: Supplementary file 6 [file crc-26-0372_supplementary_figure_s5_suppsf5.docx]

**Supplementary Figure S5.** Calibration plots of pooled overall survival (OS) predictions at 1, 2, 3, and 4 years, obtained by weighted pooling of landmark-specific predictions in the development (A-D) and validation (E-H) cohorts. For each prediction horizon, patients were subgrouped together by quantiles of the predicted OS probability. Points show the observed OS at that horizon displayed against model-predicted probability. Observed OS was estimated by the Kaplan-Meier method within each subgroup, with vertical bars indicating the corresponding 95% confidence intervals. The 45° line indicates perfect calibration; curves lying close to the diagonal denotes good agreement between predicted and observed survival.
