# Supplementary material for: Prognostic Modeling Integrating RAS and SMAD4 Status in Patients with BRAF Wild-Type, Microsatellite-Stable Metastatic Colorectal Cancer
Source: Cancer Res Commun. 2026 Nov 10;6(11):2644–54. doi: 10.1158/2767-9764.CRC-26-0372 (PMC13750249; doi:10.1158/2767-9764.CRC-26-0372)
Supplement: Supplementary file 7 [file crc-26-0372_supplementary_figure_s6_suppsf6.docx]

**Supplementary Figure S6.** Time-dependent discriminative ability measure(Area Under the Curve [AUC]) at 1-, 2-, 3-, and 4-year horizons across 11 prespecified landmark times (0-30 months, 3-month steps) in the development cohort (panels A-D) and the validation cohort (panels E-H). Each point represents the landmark-specific AUC with the corresponding 95% CI. The “Overall” diamond summarizes the pooled AUC across all landmarks, obtained by component-wise aggregation of Inverse Probability of censoring Weighted (IPCW) concordance contributions. Cases were patients who died within the prediction horizon; controls were patients alive beyond it. Uncertainty (quantified by 95%CIs) was estimated by patient-level cluster bootstrap resampling.
