# Supplementary material for: Prognostic Modeling Integrating RAS and SMAD4 Status in Patients with BRAF Wild-Type, Microsatellite-Stable Metastatic Colorectal Cancer
Source: Cancer Res Commun. 2026 Nov 10;6(11):2644–54. doi: 10.1158/2767-9764.CRC-26-0372 (PMC13750249; doi:10.1158/2767-9764.CRC-26-0372)
Supplement: Supplementary file 9 [file crc-26-0372_supplementary_figure_s8_suppsf8.docx]

**Supplementary Figure S8.** Dynamic clinic-molecular nomogram to predict overall survival probability for patients at the time of metastatic disease diagnosis (i.e., landmark time origin: month 0). Example for a 25-years old patient, with non-resected right-colon primary tumor with ECOG≥1; one metastatic site (synchronous); *RAS* wild-type and *SMAD4* alteration. The panel displays the full survival trajectory from month 0 up to 90 months and the corresponding **conditional** overall survival probabilities at 12, 24, 36, and 48 months **after metastatic diagnosis**.
