# Supplementary material for: Prognostic Modeling Integrating RAS and SMAD4 Status in Patients with BRAF Wild-Type, Microsatellite-Stable Metastatic Colorectal Cancer
Source: Cancer Res Commun. 2026 Nov 10;6(11):2644–54. doi: 10.1158/2767-9764.CRC-26-0372 (PMC13750249; doi:10.1158/2767-9764.CRC-26-0372)
Supplement: Supplementary file 10 [file crc-26-0372_supplementary_figure_s9_suppsf9.docx]

**Supplementary Figure S9.** Dynamic nomogram to predict overall survival probability for patients undergoing metastasectomy at 3 months from metastatic disease diagnosis (i.e., landmark time origin: month 3). Example for a 25-years old patient, with non-resected right-colon primary tumor with ECOG≥1; one metastatic site (synchronous); *RAS* wild-type and *SMAD4* alteration. The panel shows the survival curve from surgery up to 90 months and the **conditional** overall survival probabilities at 12, 24, 36, and 48 months **after surgery**, conditional on being alive at the time of surgery. The model explicitly accounts for the timing of metastasectomy, hence users only need to specify the month of surgery rather than selecting a separate landmark time.
