# Supplementary material for: Prognostic Modeling Integrating RAS and SMAD4 Status in Patients with BRAF Wild-Type, Microsatellite-Stable Metastatic Colorectal Cancer
Source: Cancer Res Commun. 2026 Nov 10;6(11):2644–54. doi: 10.1158/2767-9764.CRC-26-0372 (PMC13750249; doi:10.1158/2767-9764.CRC-26-0372)
Supplement: Supplementary file 11 [file crc-26-0372_supplementary_methods_suppsm.docx]

**Supplementary Methods**

**Genomic alterations**

Genomic alterations were coded as ‘1’ (presence of alteration) or ‘0’ (wild-type status), except for genes with strong literature support for distinct prognostic roles of different types of alterations including *ALK* (mutations and rearrangements were coded separately), *EGFR* (mutations and amplifications coded separately), *ERBB2* (mutations and amplifications coded separately), *FGFR1* (mutations and rearrangements coded separately), *MET* (mutations and amplifications coded separately), *NTRK1-3* (mutations and rearrangements coded separately), *RET* (mutations and rearrangements coded separately), *ROS1* (mutations and rearrangements coded separately), *PTEN* (mutations and losses coded separately). For *SMAD4*, pathogenic sequence variants and genomic losses were combined into a single gene-level alteration indicator, as both alteration classes may result in impaired SMAD4 function.

**Statistical analysis**

*Stability Selection:*

Stability selection was performed across 10,000 repetitions using an L1-penalized Cox model stratified by landmark interval (distinct baseline hazards per stratum; common regression coefficients across strata). Penalization was applied to gene variables and specific sites of metastases, whereas (prespecified) clinical variables (age, sex, ECOG PS, primary tumor sidedness, primary tumor resection, time-to-metastases, number of metastatic sites, and metastasectomy) were included unpenalized, based on prior evidence of prognostic relevance and to preserve transportability given their routine availability in clinical records, whereas tumor genomic profiling requires dedicated assays with heterogeneous uptake and access. The stability probability threshold (SpT) was set conservatively at 0.90. The per-subsample model size was capped at four, prioritizing reproducibility and enabling a conservative upper bound on the expected number of false selections (per-family error rate, PFER).

*Computation of pooled performance metrics*:

Because each patient can contribute to multiple landmark timepoints, performance measures at a given prediction horizon were summarized using an aggregation scheme that respects the stratum-specific risk sets and censoring. Hence, the pooled measure represents an overall measure at each prediction horizon, obtained by averaging the performance measures across all landmark time points (landmark strata). For the time-dependent Area Under the Curve (AUC), within each stratum we computed Inverse Probability of censoring weighted (IPCW) concordance contributions for all eligible case-control pairs and then pooled across strata by summing these contributions and normalizing by the total IPCW pair weight. For the dynamic Brier score, IPCW-weighted squared-error contributions were summed across strata and normalized by the total IPCW weight among eligible individuals. This component-wise aggregation is equivalent to evaluating the metric on the union of landmark risk sets while retaining the correct stratum-specific censoring adjustments, and it avoids the distortions of naïve averaging across strata.
