# Supplementary material for: Prognostic Modeling Integrating RAS and SMAD4 Status in Patients with BRAF Wild-Type, Microsatellite-Stable Metastatic Colorectal Cancer
Source: Cancer Res Commun. 2026 Nov 10;6(11):2644–54. doi: 10.1158/2767-9764.CRC-26-0372 (PMC13750249; doi:10.1158/2767-9764.CRC-26-0372)
Supplement: Supplementary file 12 [file crc-26-0372_supplementary_table_s1_suppst1.docx]

**Supplementary Table S1.** Frequency of genomic alterations detected by the Comprehensive Genomic Profiling (CGP). Alteration classes include single-nucleotide variants/indel, copy-number gains, homozygous loss, and gene fusions. “*RAS*” groups canonical *KRAS*/*NRAS* hotspot mutations, and “Atypical *RAS*” groups non-canonical activating variants.

| Gene | Prevalence  (%) | Gene | Prevalence (%) | Gene | Prevalence (%) | Gene | Prevalence (%) |
| --- | --- | --- | --- | --- | --- | --- | --- |
| *APC* | 85.2 | *BRCA2* | 1.5 | *ERBB4* | 0.6 | *CDKN1A* | 0.4 |
| *TP53* | 80.1 | *FGF3* | 1.5 | *NF2* | 0.6 | *CRKL* | 0.4 |
| *RAS* | 50.9 | *MAP3K1* | 1.5 | *NOTCH3* | 0.6 | *EPHB1* | 0.4 |
| *PIK3CA* | 19 | *CCND3* | 1.4 | *PAX5* | 0.6 | *FLT1* | 0.4 |
| *SMAD4* | 14.1 | *ERBB2* mut | 1.4 | *POLE* | 0.6 | *MAP2K2* | 0.4 |
| *FBXW7* | 11.6 | *MAP2K1* | 1.4 | *TSC2* | 0.6 | *MDM2* | 0.4 |
| *MYC* | 7.2 | *PARK2* | 1.4 | *XPO1* | 0.6 | *MSH3* | 0.4 |
| *SOX9* | 6.9 | *PBRM1* | 1.4 | *BARD1* | 0.5 | *NBN* | 0.4 |
| *GNAS* | 5.8 | *RBM10* | 1.4 | *CARD11* | 0.5 | *NTRK1 mut* | 0.4 |
| *ATM* | 5.3 | *CUL4A* | 1.2 | *CDKN1B* | 0.5 | *PMS2* | 0.4 |
| *BCL2L1* | 5.2 | *MLL2* | 1.2 | *CIC* | 0.5 | *PPP2R2A* | 0.4 |
| *CDK8* | 4.6 | *MTOR* | 1.2 | *EGFR mut* | 0.5 | *QKI* | 0.4 |
| *FAM123B* | 4.6 | *RAF1* | 1.2 | *EP300* | 0.5 | *RET* mut | 0.4 |
| *ERRB2* ampl | 4.4 | *SMARCA4* | 1.2 | *EPHB4* | 0.5 | *TNFAIP3* | 0.4 |
| *FLT3* | 4.3 | *BCOR* | 1.1 | *FANCA* | 0.5 | *AKT2* | 0.2 |
| *ARID1A* | 4.2 | *BRAF* class 3 | 1.1 | *FGFR2* | 0.5 | *ARAF* | 0.2 |
| *PTEN* loss | 4.1 | *CCND1* | 1.1 | *GABRA6* | 0.5 | *BAP1* | 0.2 |
| *PTEN* mut | 4.1 | *RICTOR* | 1.1 | *GATA6* | 0.5 | *CBFB* | 0.2 |
| *SRC* | 3.5 | *BRCA1* | 1 | *HGF* | 0.5 | *CCNE1* | 0.2 |
| *MAP2K4* | 3.3 | *CDK12* | 1 | *IDH1* | 0.5 | *CD22* | 0.2 |
| *ARFRP1* | 3.1 | *CDKN2A* | 1 | *KEL* | 0.5 | *CDK6* | 0.2 |
| *RAD21* | 3.1 | *ERBB3* | 1 | *MAPK1* | 0.5 | *CDKN2B* | 0.2 |
| *SMAD2* | 3.1 | *RB1* | 1 | *MET* ampl | 0.5 | *CEBPA* | 0.2 |
| Atypical *RAS* | 3 | *RNF43* | 1 | *MITF* | 0.5 | *CSF3R* | 0.2 |
| *AURKA* | 2.8 | *TERT* | 1 | *MLL* | 0.5 | *CTNNA1* | 0.2 |
| *DIS3* | 2.8 | *VEGFA* | 1 | *NT5C2* | 0.5 | *DDR1* | 0.2 |
| *DNMT3A* | 2.8 | *CREBBP* | 0.9 | *NTRK3 mut* | 0.5 | *DICER1* | 0.2 |
| *NF1* | 2.8 | *FGF14* | 0.9 | *PPP2R1A* | 0.5 | *EED* | 0.2 |
| *ZNF217* | 2.8 | *FGF19* | 0.9 | *SF3B1* | 0.5 | *FGF10* | 0.2 |
| *CCND2* | 2.7 | *FGF4* | 0.9 | *STAG2* | 0.5 | *FGF12* | 0.2 |
| *WHSC1L1* | 2.7 | *FLCN* | 0.9 | *STK11* | 0.5 | *FH* | 0.2 |
| *FGF23* | 2.6 | *CDH1* | 0.7 | *TBX3* | 0.5 | *GRM3* | 0.2 |
| *CTNNB1* | 2.5 | *CHEK2* | 0.7 | *ACVR1B* | 0.4 | *IGF1R* | 0.2 |
| *FGFR1* fusion | 2.5 | *KDM6A* | 0.7 | *ARID2* | 0.4 | *IKZF1* | 0.2 |
| *MUTYH* | 2.5 | *LYN* | 0.7 | *ATRX* | 0.4 | *IRF2* | 0.2 |
| *FGF6* | 2.2 | *NOTCH1* | 0.7 | *ACVR1B* | 0.4 | *JUN* | 0.2 |
| *PIK3R1* | 2.2 | *PTPN11* | 0.7 | *ARID2* | 0.4 | *KDR* | 0.2 |
| *ZNF703* | 2 | *RAD54L* | 0.7 | *ATRX* | 0.4 | *MCL1* | 0.2 |
| *EGFR* ampl | 1.8 | *SETD2* | 0.7 | *BCL6* | 0.4 | *MRE11A* | 0.2 |
| *ATR* | 1.6 | *TET2* | 0.7 | *BCL9* | 0.4 | *MSH6* | 0.2 |
| *IRS2* | 1.6 | *AKT1* | 0.6 | *BCORL1* | 0.4 | *NOTCH2* | 0.2 |
| *KDM5A* | 1.6 | *CASP8* | 0.6 | *BRD4* | 0.4 | *NTRK2* mut | 0.2 |
| *ASXL1* | 1.5 | *EPHA3* | 0.6 | *CDC73* | 0.4 | *PALB2* | 0.2 |
| *PDGFRA* | 0.2 | *KDM5C* | 0.1 |  |  |  |  |
| *PIK3C2G* | 0.2 | *KEAP1* | 0.1 |  |  |  |  |
| *PIK3CB* | 0.2 | *KIT* | 0.1 |  |  |  |  |
| *PIM1* | 0.2 | *KLHL6* | 0.1 |  |  |  |  |
| *PRDM1* | 0.2 | *KMT2C* | 0.1 |  |  |  |  |
| *PRKCI* | 0.2 | *MAP3K13* | 0.1 |  |  |  |  |
| *PTCH1* | 0.2 | *MAX* | 0.1 |  |  |  |  |
| *PTPRO* | 0.2 | *MEN1* | 0.1 |  |  |  |  |
| *RET* fusion | 0.2 | *MET* mut | 0.1 |  |  |  |  |
| *ROS1* mut | 0.2 | *MLH1* | 0.1 |  |  |  |  |
| *SDHA* | 0.2 | *MSH2* | 0.1 |  |  |  |  |
| *SGK1* | 0.2 | *MTAP* | 0.1 |  |  |  |  |
| *SMARCB1* | 0.2 | *MYB* | 0.1 |  |  |  |  |
| *SPEN* | 0.2 | *MYCL1* | 0.1 |  |  |  |  |
| *TGFBR2* | 0.2 | *NFKBIA* | 0.1 |  |  |  |  |
| *TNFRSF14* | 0.2 | *NKX2−1* | 0.1 |  |  |  |  |
| *TSC1* | 0.2 | *NTRK* fusions | 0.1 |  |  |  |  |
| *U2AF1* | 0.2 | *PARP1* | 0.1 |  |  |  |  |
| *VHL* | 0.2 | *PDGFRB* | 0.1 |  |  |  |  |
| *XRCC2* | 0.2 | *POLD1* | 0.1 |  |  |  |  |
| *ABL1* | 0.1 | *RAD51B* | 0.1 |  |  |  |  |
| *ALK* fusion | 0.1 | *RAD51C* | 0.1 |  |  |  |  |
| *ALK* mut | 0.1 | *RARA* | 0.1 |  |  |  |  |
| *AR* | 0.1 | *REL* | 0.1 |  |  |  |  |
| *AURKB* | 0.1 | *ROS1* fusion | 0.1 |  |  |  |  |
| *AXIN1* | 0.1 | *RPTOR* | 0.1 |  |  |  |  |
| *AXL* | 0.1 | *SDHC* | 0.1 |  |  |  |  |
| *BCL2* | 0.1 | *SLC34A2* | 0.1 |  |  |  |  |
| *BCL2L2* | 0.1 | *SMO* | 0.1 |  |  |  |  |
| *CALR* | 0.1 | *SNCAIP* | 0.1 |  |  |  |  |
| *CBL* | 0.1 | *SPOP* | 0.1 |  |  |  |  |
| *CD74* | 0.1 | *SUFU* | 0.1 |  |  |  |  |
| *CD79A* | 0.1 | *WRN* | 0.1 |  |  |  |  |
| *CHEK1* | 0.1 | *WT1* | 0.1 |  |  |  |  |
| *CUL3* | 0.1 |  |  |  |  |  |  |
| *CXCR4* | 0.1 |  |  |  |  |  |  |
| *DDR2* | 0.1 |  |  |  |  |  |  |
| *EMSY* | 0.1 |  |  |  |  |  |  |
| *ERCC2* | 0.1 |  |  |  |  |  |  |
| *ERG* | 0.1 |  |  |  |  |  |  |
| *ESR1* | 0.1 |  |  |  |  |  |  |
| *ETV6* | 0.1 |  |  |  |  |  |  |
| *EZH2* | 0.1 |  |  |  |  |  |  |
| *FANCC* | 0.1 |  |  |  |  |  |  |
| *FAS* | 0.1 |  |  |  |  |  |  |
| *GNA11* | 0.1 |  |  |  |  |  |  |
| *GNAQ* | 0.1 |  |  |  |  |  |  |
| *INPP4B* | 0.1 |  |  |  |  |  |  |
| *JAK3* | 0.1 |  |  |  |  |  |  |

List of Abbreviations. Mut: mutation. Ampl: amplification.
