# Supplementary material for: Prognostic Modeling Integrating RAS and SMAD4 Status in Patients with BRAF Wild-Type, Microsatellite-Stable Metastatic Colorectal Cancer
Source: Cancer Res Commun. 2026 Nov 10;6(11):2644–54. doi: 10.1158/2767-9764.CRC-26-0372 (PMC13750249; doi:10.1158/2767-9764.CRC-26-0372)
Supplement: Supplementary file 13 [file crc-26-0372_supplementary_table_s2_suppst2.docx]

**Supplementary Table S2.** Patients and disease’s characteristics according to *RAS* and *SMAD4* status in the overall cohort (N=1071).

| **Characteristic** | ***RAS/SMAD4* status** | | | |  |
| --- | --- | --- | --- | --- | --- |
|  | **Double WT**  **N= 466 (n, %)** | ***RAS*mut only**  **N= 455 (n, %)** | ***SMAD*4alt only**  **N= 71 (n, %)** | **Double mut**  **N= 79 (n, %)** |  |
| **Sex** |  |  |  |  |  |
| Female | 191 (41) | 219 (48) | 27 (38) | 29 (37) |  |
| Male | 275 (59) | 236 (52) | 44 (62) | 50 (63) |  |
| **Age (median, IQR)** | 61.5 [52.2-69.1] | 59.3 [50.2-66.5] | 59.5 [48.2-68.7] | 59.4 [50.2-68.1] |  |
| **ECOG PS** |  |  |  |  |  |
| 0 | 338 (74) | 379 (85) | 43 (61) | 65 (83) |  |
| ≥1 | 120 (26) | 68 (15) | 27 (39) | 13 (17) |  |
| (Missing) | 8 | 8 | 1 | 1 |  |
| **Primary Tumor Sidedness** |  |  |  |  |  |
| Left | 380 (82) | 284 (62) | 50 (70) | 44 (56) |  |
| Right | 86 (18) | 171 (38) | 21 (30) | 35 (44) |  |
| **Resection of primary tumor** |  |  |  |  |  |
| Yes | 188 (40) | 158 (35) | 24 (34) | 25 (32) |  |
| No | 278 (60) | 297 (65) | 47 (66) | 54 (68) |  |
| **Onset of Metastases** |  |  |  |  |  |
| Metachronous | 137 (29) | 98 (22) | 11 (15) | 11 (14) |  |
| Synchronous | 329 (71) | 357 (78) | 60 (85) | 68 (86) |  |
| **Number of metastatic sites** |  |  |  |  |  |
| 1 | 267 (57) | 223 (49) | 49 (69) | 40 (51) |  |
| ≥2 | 199 (43) | 232 (51) | 22 (31) | 39 (49) |  |
| **Liver metastases** |  |  |  |  |  |
| Yes | 336 (72) | 320 (70) | 49 (69) | 62 (78) |  |
| No | 130 (28) | 135 (30) | 22 (31) | 17 (22) |  |
| **Peritoneal metastases** |  |  |  |  |  |
| Yes | 75 (16) | 106 (23) | 18 (25) | 23 (29) |  |
| No | 391 (84) | 349 (77) | 53 (75) | 56 (71) |  |
| **Lung metastases** |  |  |  |  |  |
| Yes | 114 (24) | 169 (37) | 5 (7) | 16 (20) |  |
| No | 352 (76) | 286 (63) | 66 (93) | 63 (80) |  |
| **Lymph nodal metastases** |  |  |  |  |  |
| Yes | 138 (30) | 126 (28) | 18 (25) | 22 (28) |  |
| No | 328 (70) | 329 (72) | 53 (75) | 57 (72) |  |
| **R0/1 metastasectomy** |  |  |  |  |  |
| Yes | 189 (41) | 126 (28) | 28 (39) | 20 (25) |  |
| No | 277 (59) | 329 (72) | 43 (61) | 59 (75) |  |
