# Supplementary material for: Prognostic Modeling Integrating RAS and SMAD4 Status in Patients with BRAF Wild-Type, Microsatellite-Stable Metastatic Colorectal Cancer
Source: Cancer Res Commun. 2026 Nov 10;6(11):2644–54. doi: 10.1158/2767-9764.CRC-26-0372 (PMC13750249; doi:10.1158/2767-9764.CRC-26-0372)
Supplement: Supplementary file 14 [file crc-26-0372_supplementary_table_s3_suppst3.docx]

**Supplementary Table S3.** Pooled performance comparison between the clinical-only landmark Cox model and the final clinic-molecular landmark Cox model including *RAS* and *SMAD4*.

| **Cohort** | **Metric** | **Horizon** | **Clinical only**  **(95% CI)** | **Clinical + *RAS*/*SMAD4***  **(95% CI)** |
| --- | --- | --- | --- | --- |
| **Development** | Time-dependent AUC | 1 year | 0.713 (0.685-0.736) | 0.723 (0.698-0.749) |
|  |  | 2 year | 0.714 (0.681-0.741) | 0.727 (0.700-0.755) |
|  |  | 3 year | 0.711 (0.676-0.741) | 0.725 (0.692-0.755) |
|  |  | 4 year | 0.716 (0.674-0.752) | 0.736 (0.697-0.771) |
|  | Brier score | 1 year | 0.163 (0.152-0.173) | 0.156 (0.147-0.166) |
|  |  | 2 year | 0.216 (0.208-0.225) | 0.210 (0.200-0.218) |
|  |  | 3 year | 0.217 (0.207-0.228) | 0.212 (0.199-0.225) |
|  |  | 4 year | 0.193 (0.178-0.209) | 0.188 (0.171-0.207) |
| **Validation** | Time-dependent AUC | 1 year | 0.644 (0.595-0.694) | 0.657 (0.604-0.707) |
|  |  | 2 year | 0.640 (0.592-0.690) | 0.658 (0.603-0.712) |
|  |  | 3 year | 0.647 (0.583-0.702) | 0.668 (0.608-0.723) |
|  |  | 4 year | 0.696 (0.624-0.766) | 0.711 (0.633-0.772) |
|  | Brier score | 1 year | 0.175 (0.156-0.195) | 0.169 (0.154-0.186) |
|  |  | 2 year | 0.235 (0.220-0.252) | 0.229 (0.216-0.247) |
|  |  | 3 year | 0.232 (0.216-0.251) | 0.227 (0.206-0.251) |
|  |  | 4 year | 0.196 (0.170-0.218) | 0.192 (0.160-0.227) |
